# Depletion of Demethylase KDM6 Enhances Early Neuroectoderm Commitment of Human PSCs

**DOI:** 10.3389/fcell.2021.702462

**Published:** 2021-09-08

**Authors:** Yajing Meng, Tianzhe Zhang, Ran Zheng, Song Ding, Jie Yang, Ran Liu, Yingan Jiang, Wei Jiang

**Affiliations:** ^1^Department of Biological Repositories, Frontier Science Center for Immunology and Metabolism, Medical Research Institute, Zhongnan Hospital of Wuhan University, Wuhan University, Wuhan, China; ^2^Department of Infectious Diseases, Renmin Hospital of Wuhan University, Wuhan, China; ^3^Human Genetics Resource Preservation Center of Wuhan University, Wuhan, China; ^4^Hubei Provincial Key Laboratory of Developmentally Originated Disease, Wuhan, China

**Keywords:** KDM6, neuroectoderm differentiation, human embryonic stem cell, GSK-J1, KDM6A, KDM6B, histone demethylase

## Abstract

Epigenetic modifications play a crucial role in neurogenesis, learning, and memory, but the study of their role in early neuroectoderm commitment from pluripotent inner cell mass is relatively lacking. Here we utilized the system of directed neuroectoderm differentiation from human embryonic stem cells and identified that KDM6B, an enzyme responsible to erase H3K27me3, was the most upregulated enzyme of histone methylation during neuroectoderm differentiation by transcriptome analysis. We then constructed KDM6B-null embryonic stem cells and found strikingly that the pluripotent stem cells with KDM6B knockout exhibited much higher neuroectoderm induction efficiency. Furthermore, we constructed a series of embryonic stem cell lines knocking out the other H3K27 demethylase KDM6A, and depleting both KDM6A and KDM6B, respectively. These cell lines together confirmed that KDM6 impeded early neuroectoderm commitment. By RNA-seq, we found that the expression levels of a panel of WNT genes were significantly affected upon depletion of KDM6. Importantly, the result that WNT agonist and antagonist could abolish the differential neuroectoderm induction due to manipulating KDM6 further demonstrated that WNT was the major downstream of KDM6 during early neural induction. Moreover, we found that the chemical GSK-J1, an inhibitor of KDM6, could enhance neuroectoderm induction from both embryonic stem cells and induced pluripotent stem cells. Taken together, our findings not only illustrated the important role of the histone methylation modifier KDM6 in early neurogenesis, providing insights into the precise epigenetic regulation in cell fate determination, but also showed that the inhibitor of KDM6 could facilitate neuroectoderm differentiation from human pluripotent stem cells.

## Introduction

Cell fate determination involves a series of precise regulation of temporal and spatial gene expression. Epigenetic changes, including histone modification, DNA methylation, and chromatin remodeling, are critically required for the generation of cell diversity during development (Podobinska et al., 2017). Among the histone tail modifications, trimethylation of lysine 27 on histone 3 (H3K27me3) and trimethylation of lysine 4 on histone 3 (H3K4me3), which play an important role in gene repression and activation, respectively, are reported to be deposited in the promoters of developmental genes in embryonic stem cells (ESCs), well known as the bivalent state (Bernstein et al., 2006). H3K27me3 is established by polycomb repressive complex 2 (PRC2) and erased by the KDM6 family (Laugesen et al., 2019; Tran et al., 2020). KDM6 family members include catalytically active KDM6A (also UTX) and KDM6B (also JMJD3) sharing a well-conserved JmjC histone demethylation domain, while KDM6C (also UTY) is considered with no catalytic activity (Sengoku and Yokoyama, 2011; Jones et al., 2018; Gazova et al., 2019). Loss-of-function of *Kdm6b* in mouse leads to postnatal lethality due to a defect in the embryonic respiratory neuronal network (Burgold et al., 2012). Mouse with Kdm6a knockout exhibits slightly different phenotype between male and female: the female Kdm6a-knockout mouse shows higher mortality rate and exhibited anxiety-like behaviors than male (Shpargel et al., 2012; Tang et al., 2017).

Neuroectoderm commitment is the first step to generate functional neurons from pluripotent stem cells (PSCs), which brings a great promise to treat degenerative neurological diseases and central nervous system injury (Kim et al., 2020). Zhang et al. (2001) first reported in 2001 that transplantable neural precursors could be generated from human embryonic stem cells (ESCs). They induced H1 and H9 cell lines into a mixture of neurons, astrocytes, and oligodendrocytes cultured with FGF-2 through *in vitro* embryoid body strategy (Zhang et al., 2001). In 2009, Chambers et al. (2009) developed an efficient protocol using Noggin and SB431542 to, respectively, inhibit BMP and TGFβ/Activin/Nodal signaling pathways, by which they could induce neural progenitor cells (NPCs) from human ESCs and induced pluripotent stem cells (iPSCs) in monolayer culture. Later, the same group replaced the growth factor Noggin with chemical molecule LDN193189 in a Matrigel-cultured system (Chambers et al., 2011) to even better generate NPCs, which later was widely applied in many other studies (Chailangkarn et al., 2016). In 2012, Nakano et al. (2012) used a 3D stem cell culture technique to get retinal tissues from human ESCs. More and more protocols then have been developed for either generating functional specific neuron or modeling neural disease, but the underlying epigenetic regulation mechanism, particularly histone methylation, has just been revealed. For instance, H3K9 methyltransferase SETDB1 was reported to play an important role in Huntington’s disease since Huntingtin protein could bind ATF7IP and competitively interfered the SETDB1 activity as a heterochromatin regulator and transcriptional repressor to maintain the low level of H3K9 trimethylation in human ESCs. Depletion of Huntingtin protein resulted in reduced neural differentiation, which phenocopied the Huntington patients’ iPSCs (Irmak et al., 2018). H3K4 demethylase KDM5B variants caused a syndrome with intellectual disability, and patients with its mutations showed severe developmental delay without dystonia until mid-childhood (Faundes et al., 2018). In addition, PRC2 complex possessed the activity of H3K27 methyltransferase, which contained SUZ12, EZH2, and EED, and the mutations of SUZ12, EZH2, and EED were found in Weaver syndrome and Weaver-like syndrome, an overgrowth/intellectual disability syndrome (Imagawa et al., 2017, 2018). However, whether other histone methylation or demethylation functions in human neurogenesis and related diseases, particularly in the process of early neuroectoderm commitment, is still poorly understood.

Here, we utilized the well-established protocol of neuroectoderm differentiation from human ESCs and analyzed the expression dynamics of enzymes responsible to histone methylations. We found that KDM6B exhibited the most significantly differential expression and thus generated a series of KDM6-knockout ESC lines to investigate the biological role and the downstream mechanism of KDM6 in human neuroectoderm commitment.

## Materials and Methods

### ESC Culture and Differentiation

Human ESC line HUES8 and iPSC line WTC (Miyaoka et al., 2014) were cultured in Matrigel-coated plates (MATRIGEL MATRIX HESC-QUALIFIED, BD Bioscience, #354277) with mTeSR1 medium (Stemcell Technologies, #1000023391) and passaged with accutase (Stemcell Technologies, #A1110501) every 3–4 days when the density achieved around 80–90% and cell cryopreservation by CS10 (Stemcell, #7930).

For neuroectoderm differentiation, ESCs or iPSCs were passaged at the density of 2 × 10^5^ cells per well for a 24-well Matrigel-coated plate (MATRIGEL MATRIX GFR, BD Bioscience, #354230). After 2 days of culture in mTeSR1, ESCs were then treated with N2B27 medium: KnockOut^TM^ DMEM/F12 (Gibco, #12660012), 0.5x N2 (Shanghai BasalMedia Technologies, #M430721), 0.5x B27 without vitamin A (Shanghai BasalMedia Technologies, #B430805), 10% Penicillin-Streptomycin (Thermo Fisher Scientific, #15140163), 1% 2-Mercaptoethanol (Gibco, #2121115), 1% GlutaMAX^TM^ Supplement (Thermo Fisher Scientific, #35050061), 1% MEM Non-Essential Amino Acids (Gibco, #11140-050), plus 2.5 μM SB431542 (Selleck, #S1067), and 0.05 μM LDN193189 (Selleck, #S7507) for 4 days, and then treated with N2B27 medium plus 0.05 μM LDN for another 2 or more days. For a WNT signal agonist and inhibitor, we used CHIR (Selleck, #S1180) or IWR1 (Selleck, #S2924); for a KDM6 inhibitor, we added GSKJ1 (Selleck, #S7581) and the control GSK126 targeting EZH2 (Selleck, #S7061).

For neuron differentiation, after neuroectoderm differentiation, we further added BDNF (MCE, #HY-P7116A), ascorbic acid (Sigma, #A4544), and FGF2 (PeproTech, #100-18B) for another 2 weeks.

For definitive endoderm differentiation, the endoderm differentiation medium [50% IMDM and 50% F12, supplemented with 0.5% B27 without vitamin A, 0.2% BSA (YEASEN, #B57370), and 100 ng/ml activin A (PeproTech, # 120-14P)] was applied for 3 days (Yang et al., 2020).

Hanging drop is used for embryoid body (EB) assay. Briefly, one drop containing 100 single ESCs was prepared and cultured with mTeSR1. On the next day, these formed EBs were transferred into culture dish with EB medium (DF12 plus 10% FBS) for another 6 days. The medium was changed every day, and we collected the EBs on day 7 to determine the gene expression.

### Generation of KDM6-Knockout ESC Lines by CRISPR/Cas9

We designed the sequences of RNA guides according to the website^1^ and the genomic screening (Shalem et al., 2014; Wang et al., 2014). We verified the cutting efficiency by a pCAG-EGxxFP reporter (Fujihara and Ikawa, 2014), and the sequences of guides used in the present work are listed as follows—KDM6A KO guide1: CCTGGGAGATAAAGCCACCA; KDM6A KO guide2: ATCCTAATTCTGGCCAGTCC; KDM6B KO guide1: AGGCTGGATGCATCGGGCAG; KDM6B KO guide2: CCGCATTGGCCGACTGCAGC.

The individual guide sequence was cloned into a pX459 vector, and then two plasmids containing two different guides were electroporated into HUES8 cells using Nucleofector (Lonza). After 2 days of selection with puromycin (Santa-cruz, #sc-108071B), the survived cells were maintained in mTeSR1 and colonized. Usually, 20–30 colonies were obtained for genotyping using primers for KDM6A-EXON10 (TAATGGCCAGAATTGGCAGT/GAAACGTCCTGCTTAG ACCAGAT) and KDM6B-EXON4/6 (AGAATTGGCTGT GAAAGGACTG/GAGAGAAGAGAAAATGGCACGGG). Those colonies with correct sequences interfering KDM6 coding were picked and then expanded for further functional analysis. The double knockout of KDM6A and KDM6B was made based on two different KDM6B-knockout clones.

### RNA Isolation and Quantitative Reverse Transcription PCR

RNA extraction and purification were performed using a HiPure Total RNA Mini Kit (Magen, #R4111-03). RNA concentration was measured using NanoDrop (Thermo Fisher Scientific). RNA was reversely transcribed into cDNA using the All-in-One cDNA Synthesis SuperMix (Bimake, #B24408). Real-time qPCR was performed with the 2x SYBR Green qPCR Master Mix (Biomake, #B21203) on the CFX384 Touch Real-Time PCR Detection System. All the mRNA reaction data were analyzed by the 2^–*dd*(Ct)^ method. All experiments were repeated at least in triplicate.

The primers used in RT-qPCR are listed as follows: *GAPDH (*AATGAAGGGGTCATTGATGG/AAGGTGAAGGTCGGAGT CAA*), OCT4 (*CAAAGCAGAAACCCTCGTGC/TCTCACTCGG TTCTCGATACTG*), NANOG (*CCCCAGCCTTTACTCTTCC TA/CCAGGTTGAATTGTTCCAGGTC*), SOX2 (*GTCATTTGC TGTGGGTGATG/AGAAAAACGAGGGAAATGGG*), SOX1 (*ATTATTTTGCCCGTTTTCCC/TCAAGGAAACACAATCGC TG*), NESTIN (*CTGCTACCCTTGAGACACCTG/TCTCTGCAT CTACGGGCTCTGA*), PAX6 (*CCGTTGGAACTGATGGAGT/G TTGGTATCCGGGGACTTC*), EOMES* (CACATTGTAGTGG GCAGTGG/CGCCACCAAACTGAGATGAT), *MIXL1* (GAG ACTTGGCACGCCTGT/GGTACCCCGACATCCACTT), *FOXA2* (GGAGCAGCTACTATGCAGAGC/CGTGTTCATGCC GTTCATCC), *SOX17* (GCATGACTCCGGTGTGAATCT/TCAC ACGTCAGGATAGTTGCAGT), *WNT3* (GGAGAGGGACCTG GTCTACTA/CTTGTGCCAAAGGAACCCGT), and *FZD5* (CA TGCCCAACCAGTTCAACC/CGGCGAGCATTGGATCTCC).

### RNA Sequencing and Data Analysis

RNA sequencing was performed in Illumina novaseq6000. Nuclei acid concentrations were measured by NanoDrop (Thermo Fisher Scientific) and Qubit 2.0 (Invitrogen) instruments. We performed RNA sequencing of two or three biological replicates for each stage/cell type at Annoroad Gene Technology. RNA sequencing reads were mapped to the *Homo sapiens* hg38 reference assembly using Hisat2, and raw counts were computed using FeatureCounts. For subsequent analysis of gene expression, genes were retained in both datasets if they were expressed in at least one sample using a TPM > 1 threshold.

Differential expression analysis was performed by DESeq2 using the raw counts generated by FeatureCounts, and genes with abs (log2 fold-change) > 1 and *p*-adjust < 0.05 were considered as significant. In order to identify the KDM6-dependent genes, we added the conditional effect (D7/D0 and D3/D0) to the differential analysis and identified the differentially expressed genes during the differentiation process. GO terms for biological process analysis of differentially expressed genes were performed by THE GENE ONTOLOGY RESOURCE website bioinformatics tool,^2^ and KEGG enrichment was performed by the ClusterProfiler R package. Heatmaps were generated using the pheat-map package in the R software.

Principal components analysis was performed using the prcomp function in the R stats package based on the covariance matrix. log_2_(TPM) of all protein coding genes was used for principal component analysis, and then we calculated and ranked the covariance of each gene for each principal component and selected the top 300 genes that contributed the most to the direction for GO enrichment.

### Immunofluorescence and AP Staining

The cultured cells were washed twice with PBS (Gibco, #C14190500BT) and fixed with 4% paraformaldehyde for 30 min at room temperature. After PBS washing for three times, blocking solution (10% donkey serum, 3‰ Triton X-100) was added for 1 h at room temperature. Then, the cells were incubated with proper primary antibodies (1:200) overnight at 4°C. After PBS washing for three times, the secondary antibodies (1:200) were added for 1 h at room temperature in dark. DAPI was finally incubated at room temperature for 10 min to counterstain the nucleus. Images were taken with a fluorescence microscope (Olympus). The information of antibodies used in this study included the following: OCT3/4(C-10) (SANTA CRUZ, #sc-5279), NANOG (M-149) (SANTA CRUZ, #SC-33760), SOX2 (Millipore, #AB5603), SOX1 (CST, #4194S), NESTIN (G-20) (SANTA CRUZ, #sc-21248), NESTIN (10c2) (SANTA CRUZ, #sc-23927), PAX6 (Biolegend, #901301), H3K27me3 (ABclonal, #A2363), synaptophysin (Sigma, #SAB4502906), NeuN (Abcam, #ab177487), β-III-tubulin (Sigma, #T8660), FOXA2 (HuaBio, #ET1703-76), TRITC-conjugated Donkey anti-rabbit IgG (Jackson Immuno Research, #711-025-152), 488-conjugated Donkey anti-Mouse IgG (Jackson Immuno Research, #715-545-150), and TRITC-conjugated Donkey anti-Mouse IgG (Jackson immuno Research, #715-025-150).

AP staining was performed by a BCIP/NBT Alkaline Phosphatase Color Development Kit (Beyotime Biotechnology, #C3206).

### Western Blot

Cell lysate was prepared on the ice by adding RIPA lysis buffer (Beyotime, P0013F) containing protease inhibitor cocktail (Roche, #4693132001). SDS-PAGE was run using 10% gel, and then proteins were transferred into nitrocellulose filter membrane (NC, Millipore, #HATF00010). The membrane was blocked with 5% evaporated skimmed milk and then incubated with primary antibodies against active beta-Catenin (Non-phosphorylated Ser33/37/Tr41) (CST, #8814), or beta-catenin (CST, #8480), or GAPDH (Proteintech, #60004-l-lg) as loading control. After overnight incubation and washing with TBST, the secondary antibody [AffiniPure Goat Anti-Mouse IgG (H + L), #AB_2338447; AffiniPure Goat Anti-Rabbit IgG (H + L), #AB_2337913] was accordingly added for 1 h at room temperature. At last, ECL mix (Immobilon ECL Ultra Western HRP Substrate, Millipore, #WBULS0100) was treated, and the membrane was visualized in the dark room.

### Statistical Analysis

RT-qPCR was analyzed by the 2^–*dd*(Ct)^ method, and immunofluorescence staining was counted by image J to get their relative fluorescence intensity. The statistical analysis was performed using Student’s *t*-test to determine the *p*-value.

### Data and Code Availability

The data presented in the study are deposited in the GEO repository, accession number (GSE172312).

## Results

### Expression Profile of Enzymes Responsible for Histone Methylation During Neuroectoderm Differentiation

**FIGURE 1:**
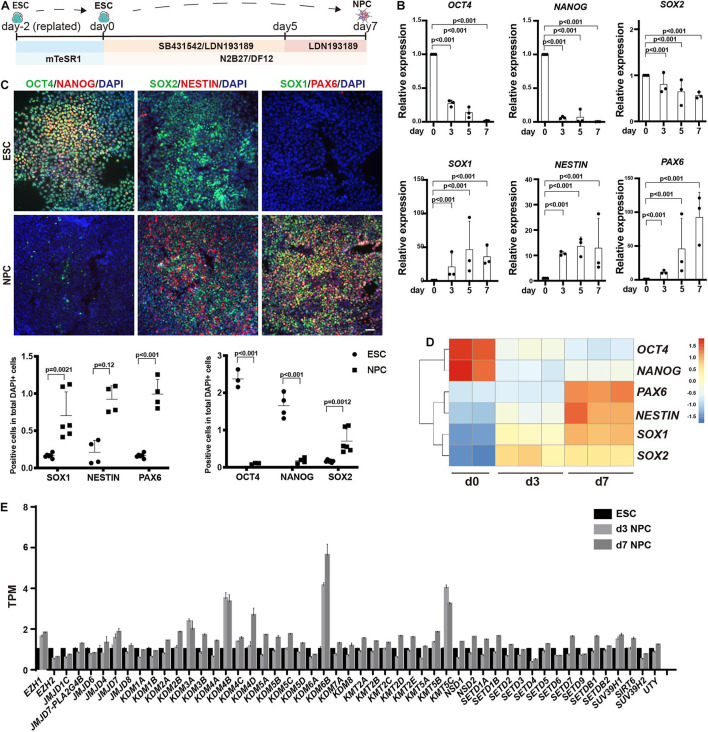
Profiling of histone methylation modifiers during human neuroectoderm differentiation. **(A)** Scheme for *in vitro* neuroectoderm differentiation. **(B,C)** RT-qPCR analysis **(B)** and immunofluorescence staining **(C)** of expression dynamics of the pluripotent or neural markers during neuroectoderm differentiation. Representative images and data statistics were shown. Day 0 means undifferentiated ESCs, and day 7 means differentiated neuroectoderm cells. Scale bar = 50 μm. **(D)** Heatmap of representative markers by RNA sequencing. **(E)** The dynamic expression patterns of histone methylation related enzymes during neuroectoderm differentiation by RNA sequencing.

We differentiated human ESCs to neuroectoderm lineage in 7 days by adding SB431542 and LDN193189 (Figure 1A). Differentiation was monitored using RT-qPCR and immunofluorescence against the pluripotent markers OCT4 and NANOG, the neuroectodermal markers SOX1, NESTIN, and PAX6, and the dual marker SOX2 (Figures 1B,C). The results showed that the RNA levels of *OCT4* and *NANOG* were downregulated dramatically during the differentiation, and the RNA levels of *SOX2* were downregulated to almost half. In addition, the RNA levels of *SOX1*, *NESTIN*, and *PAX6* were significantly upregulated, with 80 times for *SOX1*, 15 times for *NESTIN*, and 100 times for *PAX6* (Figure 1B). Consistently, the immunofluorescence assay showed dramatic decrease in OCT4 and NANOG, while the SOX1, NESTIN, and PAX6 exhibited significantly positive staining in differentiated cells (Figure 1C).

We next performed RNA-seq analysis for neuroectoderm differentiation by using undifferentiated cells, intermediate (sample at differentiation day 3), and differentiated neuroectoderm progenitors. To confirm the reliability of our RNA-seq data, we checked the expression dynamics of pluripotent genes and neural lineage genes, and the results showed that *OCT4* and *NANOG* were obviously downregulated while *SOX1*, *NESTIN*, and *PAX6* were dramatically upregulated (Figure 1D), indicating efficient neuroectoderm differentiation. Then, we surveyed the expression profile of enzymes responsible for histone methylation during neuroectoderm differentiation. Among the methyltransferases and demethylases, H3K27 demethylase *KDM6B* exhibited the highest upregulation (Figure 1E). We also checked some publicly available expression data set on neuroectoderm differentiation, and the result from LIBD Stem Cell Browser based on multiple differentiation conditions and cell lines^3^ consistently supported that KDM6B was significantly upregulated during neural progenitor differentiation (Supplementary Figure 1).

### Knockout of KDM6B Promoted the Differentiation of Neuroectoderm

**FIGURE 2:**
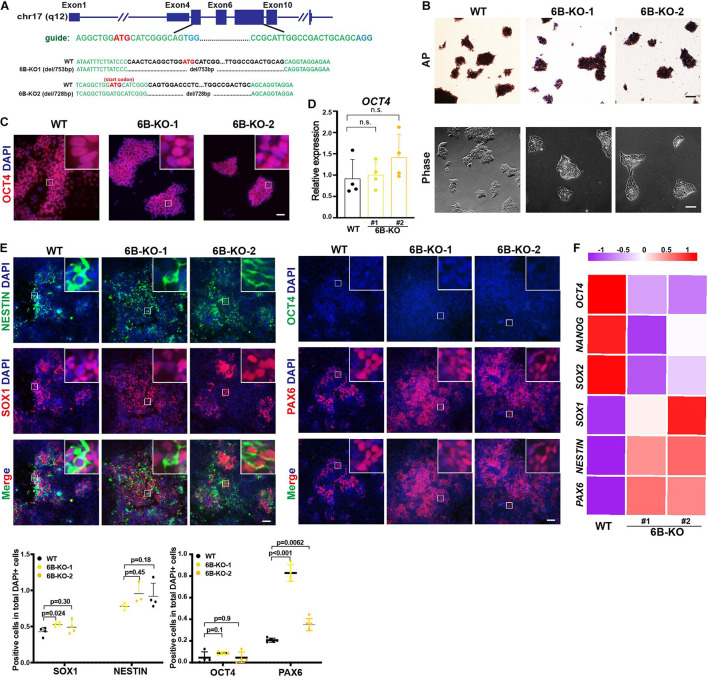
KDM6B knockout enhanced neuroectoderm differentiation. **(A)** Illustration of *KDM6B* locus, sgRNA sequences, and generated mutations. **(B)** Morphology and AP staining of KDM6B-KO cells and wild-type ESCs. Scale bar = 100 μm. **(C,D)** The immunofluorescence staining **(C)** and RT-qPCR analysis **(D)** of the pluripotent marker OCT4 in the KDM6B-KO cells at undifferentiated ESC states. Scale bar = 50 μm. **(E,F)** The immunofluorescence staining images (up) and quantification (down) **(E)** and RNA analysis **(F)** of pluripotent or neural markers in the KDM6B-KO cells after 7 days of neuroectoderm differentiation. Scale bar = 50 μm.

To investigate the biological function of KDM6B in neuroectoderm differentiation, we established KDM6B-knockout human ESC lines using CRISPR/Cas9 technology (Figure 2A). Two clones were obtained with 753 bp deletion (6B-KO1) and 728 bp deletion (6B-KO2) in exon 4 to exon 6 of KDM6B locus, respectively (Figure 2A), with typical colony morphology and positive staining of alkaline phosphatase, the same as the parental wild-type ESC HUES8 (Figure 2B). In addition, the expression levels of the pluripotent marker *OCT4* were comparable with wild-type ESCs (Figures 2C,D), indicating that KDM6B was not required for human ESC maintenance, which was consistent with our previous report by shRNA-mediated depletion of KDM6B (Jiang et al., 2013).

Then we determined the neuroectoderm differentiation potential of KDM6B-KO ESCs. We performed immunofluorescence assay after 7 days of differentiation and found out that the fluorescence intensity of neural progenitor markers SOX1 and PAX6 were significantly higher than those of the wild type despite no obvious differences of NESTIN and the pluripotent marker OCT4 (Figure 2E), indicating an enhanced neuroectoderm differentiation, which was further supported by the RNA expression analysis of key pluripotent and neuroectodermal markers (Figure 2F).

### Knockout of KDM6A Also Promoted the Differentiation of Neuroectoderm

**FIGURE 3:**
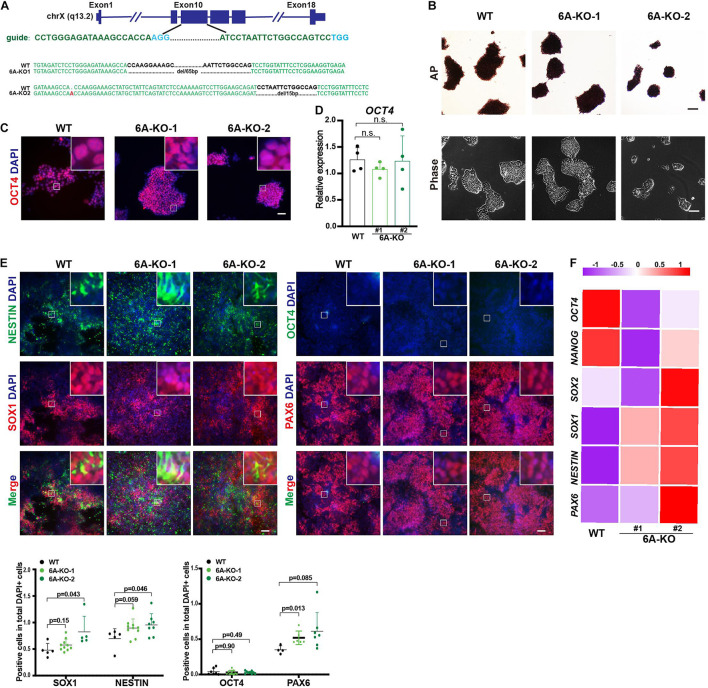
KDM6A knockout improved the neuroectoderm differentiation. **(A)** Illustration of *KDM6A* locus, sgRNA sequences, and generated mutations. **(B)** Morphology and AP staining of KDM6A-KO cells and wild-type ESCs. Scale bar = 100 μm. **(C,D)** The RT-qPCR analysis **(C)** and immunofluorescence staining **(D)** of the pluripotent marker in the KDM6A-KO cells at undifferentiated ESC states. Scale bar = 50 μm. **(E,F)** The immunofluorescence staining images (up) and quantification (down) **(E)** and RNA analysis **(F)** of pluripotent or neural markers in the KDM6A-KO cells after 7 days of neuroectoderm differentiation (*n* ≥ 3; mean ± SEM is plotted). Scale bar = 50 μm.

To determine whether demethylation activity is included in neuroectoderm differentiation, we also investigated the role of KDM6A, the other H3K27me3 demethylase that is located in the X chromosome, in neuroectoderm differentiation. We established KDM6A-knockout human ESC lines using CRISPR/Cas9 technology as well (Figure 3A). Two clones were obtained with 65 bp deletion (6A-KO1) and 1 bp insert/15 bp deletion (6A-KO2) in exon 10 of the KDM6A locus (Figure 3A), which also exhibited typical HUES8 morphology and positive staining of alkaline phosphatase (Figure 3B). The expression levels of the pluripotent marker OCT4 were comparable with wild-type ESCs as well (Figures 3C,D). Then, we subjected the KDM6A-KO ESCs to neuroectoderm differentiation. The immunofluorescence assay showed that the fluorescence intensity of neural progenitor markers NESTIN, SOX1, and PAX6 in KDM6A-KO cells exhibited higher positive percentage than wild-type cells, while the pluripotent marker OCT4 showed no significant change at day 7 (Figure 3E). We further performed the RT-qPCR assay to determine the expression levels of key pluripotent and neuroectodermal markers, and the result consistently supported higher neuroectoderm differentiation (Figure 3F). Therefore, the similar phenotype of enhanced neuroectoderm differentiation in both KDM6A-KO and KDM6B-KO cells suggested the critical role of H3K27 demethylase in human neuroectoderm differentiation.

### Double Knockout of KDM6A and KDM6B Further Promoted Neuroectoderm Differentiation

**FIGURE 4:**
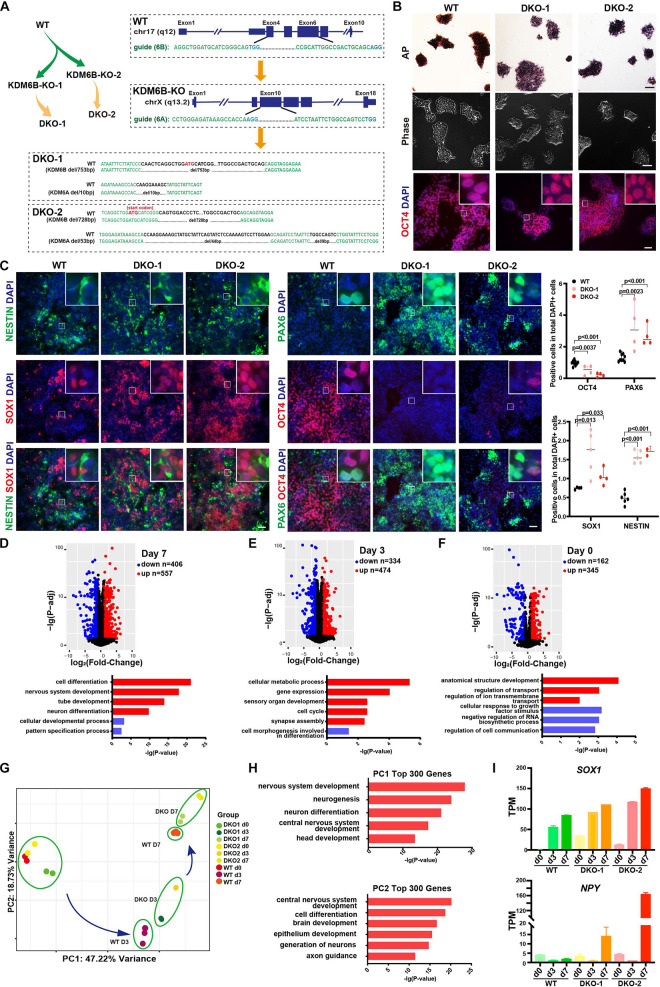
The double knockout of KDM6A and KDM6B promoted the neuroectoderm differentiation. **(A)** Illustration of the strategy to make KDM6 double-knockout cells. The genotypes of two double knockouts at *KDM6A* and *KDM6B* loci are shown. **(B)** The AP staining, morphology, and immunofluorescence staining of the pluripotent marker OCT4 in the KDM6-DKO and wild-type cells at undifferentiated ESC states. The scale bar for AP and morphology is 100 μm, while the scale bar for OCT4 staining is 50 μm. **(C)** The immunofluorescence staining images (left) and quantification (right) of pluripotent and neural markers in the KDM6-DKO cells after 7 days of neuroectoderm differentiation. Scale bar = 50 μm. **(D–F)** GO analysis of the differentially expressed genes between KDM6-DKO and wild-type cells at differentiation day 7 **(D)**, day 3 **(E)**, and day 0 **(F)**. **(G)** PCA analysis of the KDM6-DKO and wild-type cells at different stages based on RNA-seq data. **(H)** GO analysis of top 300 genes for PC1 and PC2 in **(G)** based on RNA-seq data. **(I)** The expression level of *SOX1* (representing PC1) and *NPY* (representing PC2) at different stages in KDM6-DKO and wild-type cells.

Considering the possible compensation when deleting KDM6A or KDM6B only, we further established KDM6A and KDM6B double-knockout human ESC lines based on KDM6B-KO1 and KDM6B-KO2 cells (Figure 4A). We generated two successful clones: DKO1 had 10 bp deletion of *KDM6A* and 753 bp deletion of *KDM6B*, and DKO2 had 53 bp deletion of *KDM6A* and 728 bp deletion of *KDM6B*. Then we checked the morphology and performed AP staining and found that KDM6-DKO ESCs exhibited similar characteristics to wild type (Figure 4B). We also examined the staining of the pluripotent marker OCT4 and found that it was comparable with wild-type ESCs (Figure 4B). These results together indicated that KDM6A and KDM6B were not essential for ESC maintenance. Next, we evaluated the neuroectoderm differentiation potential of KDM6-DKO ESCs. After differentiation, immunofluorescence result confirmed that NESTIN, SOX1, and PAX6 in DKO cells exhibited much higher expression than wild-type cells, and the pluripotent marker OCT4 showed significantly lower expression (Figure 4C), indicating a significantly enhanced neuroectoderm differentiation phenotype.

To better evaluate the phenotype, we performed an RNA sequencing experiment using the samples at day 0, day 3, and day 7 during neuroectodermal differentiation from KDM6-DKO and wild-type groups. The gene ontology (GO) analysis based on differential expression genes at day 7 revealed that the upregulated genes were enriched in neural differentiation related terms (Figure 4D). Similar results could be obtained from the day 3 samples as well (Figure 4E), although not many GO terms were enriched for day 0 samples (Figure 4F). Principal component analysis (PCA) of transcriptomes showed that the differentiation of DKO cells was more advanced than that of the wild type at both day 3 and day 7 (Figure 4G). We picked the top 300 genes with the most contribution to the PC1 and PC2 and found that PC1 genes are enriched in terms of nervous system development and neurogenesis, and PC2 genes are enriched in terms of cell differentiation, brain development, and axon guidance. SOX1 representing for PC1 genes and NPY representing for PC2 genes are shown, respectively (Figure 4H). Taking *SOX1* and NPY as the quantification marker of neurogenesis and neuronal differentiation, we could also observe that neural differentiation was promoted in KDM6-DKO (Figure 4I).

### ESCs With KDM6A/KDM6B Double Knockout Maintain the Multilineage Differentiation Capacity

**FIGURE 5:**
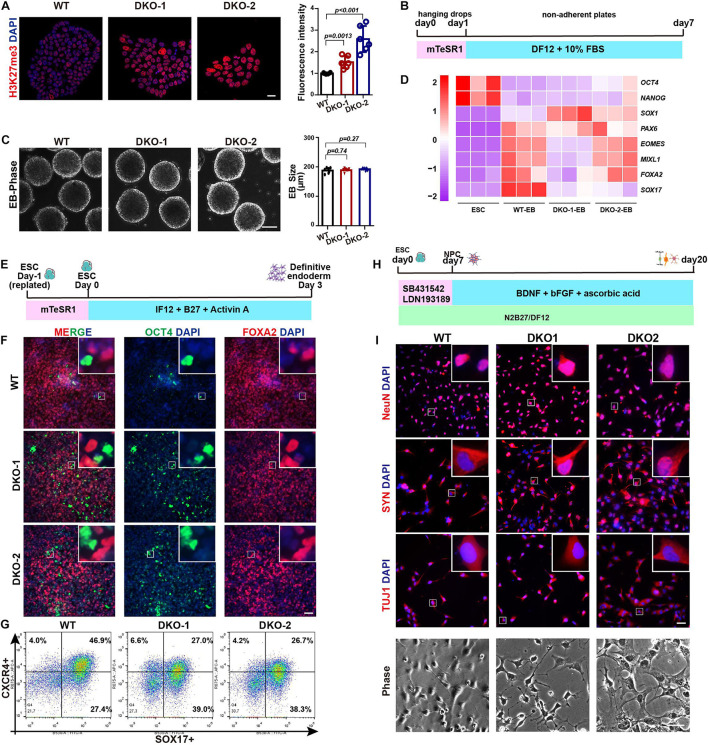
The effect of double knockout of KDM6A and KDM6B on lineage differentiation. **(A)** The immunofluorescence staining and quantification of the H3K27me3 level in the KDM6-DKO and wild-type cells. Scale bar = 20 μm. **(B)** Illustration of EB assay. **(C)** Morphology and size measurement of KDM6-DKO cells and wild-type EBs. Scale bar = 100 μm. **(D)** RNA analysis of pluripotent and lineage markers of KDM6-DKO cells and wild-type EBs. **(E)** Illustration of definitive endoderm differentiation assay. **(F,G)** The immunofluorescence staining **(F)** and flow cytometric analysis **(G)** of endoderm markers in the KDM6-DKO and wild-type cells after 3 days of endoderm induction. Scale bar = 50 μm. **(H)** Illustration of neuronal differentiation assay. **(I)** The immunofluorescence staining of neuronal markers and morphology in the KDM6-DKO and wild-type cells after 3 weeks of neuronal induction. Scale bar = 50 μm.

We further evaluated the multilineage differentiation ability of KDM6 double-knockout ESCs. We first checked the H3K27me3 level by immunostaining, and the result showed that the knockout cells exhibited much higher H3K27me3 than wild-type cells (Figure 5A), indicating the successful knockout of KDM6. We then subjected these cells to EB differentiation (Figure 5B). After 7 days of spontaneous differentiation, both knockout and wild-type ESCs could form spheres with comparable size (Figure 5C). Moreover, mRNA expression analysis indicated that both knockout and wild-type EBs highly expressed lineage markers, while the pluripotent markers were downregulated compared to undifferentiated ESCs (Figure 5D). We further performed directed definitive endoderm differentiation assay (Figure 5E; Yang et al., 2020). After 3 days of treatment with Activin A, KDM6-knockout ESCs could be induced to FOXA2-positive and OCT-negative endoderm cells by immunofluorescence examination (Figure 5F); flow cytometric analysis based on endoderm markers CXCR4 and SOX17 indicated that KDM6-knockout ESCs kept the endoderm differentiation capacity, although much lower than wild-type ESCs (Figure 5G), which was consistent with a previous study (Jiang et al., 2013). These data together excluded the possibility that KDM6-knockout ESCs lost the ability to differentiate into other lineages.

Since KDM6-knockout ESCs exhibit higher neuroectoderm differentiation capacity, we were wondering whether the generation of neuron was affected. Therefore, we utilized published protocol to subject these neuroectoderm progenitors to neuronal differentiation by adding BDNF, ascorbic acid, and FGF2 for another 2 weeks (Figure 5H; Zhang et al., 2001; Topol et al., 2015). We found that the KDM6-knockout cells could generate neurons with typical morphology and positively expressing NeuN, synaptophysin (SYN), and β-III-tubulin (TUJ1) (Figure 5I), similar to wild-type cells.

### WNT Signal Was Involved in KDM6-Mediated Neuroectoderm Differentiation

**FIGURE 6:**
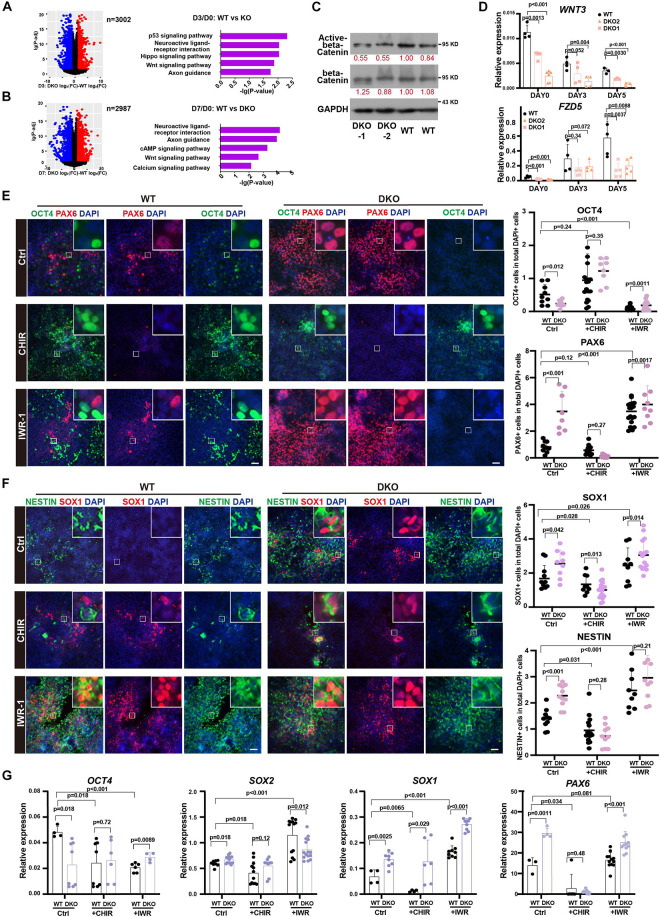
WNT signal pathway contributed to the function of KDM6 during neuroectoderm differentiation. **(A,B)** KEGG analysis of abnormal activation or repression during neuroectoderm differentiation caused by KDM6 knockout based on conditional effect differential expression analysis at differentiation day 3 **(A)** and day 7 **(B)** over undifferentiated state. **(C)** Western blot assay showing the signal intensity of active beta-catenin and total beta-catenin in KDM6-DKO cells and wild-type cells. GAPDH works as the loading control. **(D)** The RT-qPCR test of *WNT3* and *FZD5* at different days during neuroectoderm differentiation. **(E,F)** The immunofluorescence test (left) and its counts (right) of the expression differences of pluripotential and NPC markers (OCT4/PAX6, NESTIN/SOX1) of HUES8 and DKO cells upon adding CHIR and IWR-1 or vehicle control. Scale bar = 50 μm. **(G)** The RT-qPCR test of the expression differences of pluripotential and neural markers of HUES8 and DKO cells upon adding CHIR or vehicle control.

Next, to study the downstream signals affected by KDM6, we surveyed the affected gene expression dynamics during neuroectoderm differentiation. We identified those genes showing abnormal activation or inhibition during differentiation by comparing the fold change of the expression level between the neural progenitor and the undifferentiated state (Figures 6A,B). We next performed KEGG enrichment analysis of these genes with abnormal expression change. Interestingly, the WNT signal pathway was significantly enriched in both day 3 and day 7 samples compared with the undifferentiated state (Figures 6A,B). This finding prompted us to look into the effect of WNT activity upon KDM6 knockout. Since non-phosphorylated beta-catenin (Ser33/37/Thr41) represents a nuclear-bound activated form because phosphorylation of beta-catenin at serine 37 and serine 33 by GSK3 would lead to its ubiquitination and degradation (Kimelman and Xu, 2006), we checked the signal of active beta-catenin to determine the WNT/beta-catenin activity. We observed a lower signal of active beta-catenin in KDM6-knockout cells, indicating that KDM6-knockout cells exhibited lower WNT activity (Figure 6C). To confirm that WNT activity was indeed impaired by KDM6 knockout, we further performed RT-qPCR assay for *WNT3* and *FZD5*, which are well-known WNT target genes and positively regulating WNT activity as well. The results showed that both *WNT3* and *FZD5* exhibited significantly lower expression in KDM6 knockout samples than the wild type (Figure 6D).

Since human ESCs with lower activity of the WNT signaling pathway could generate primarily neuroectodermal cells (Blauwkamp et al., 2012), and CHIR-99021 (CHIR for short), a WNT agonist, has been reported to block early neural differentiation (Moya et al., 2014), we suspected that the WNT signal pathway might be the functional downstream of KDM6 to promote neuroectoderm differentiation. To test this hypothesis, we applied WNT antagonist IWR-1 and agonist CHIR in the neuroectoderm differentiation of KDM6-DKO cells. The result showed that CHIR blocked the expression of neuroectoderm markers (NESTIN, SOX1, and PAX6) in both wild-type and KDM6-DKO cells determined by immunofluorescence, making no significant difference between the two genotypes (Figures 6E,F); meanwhile, the IWR-1 treatment improved the expression of neuroectoderm markers (NESTIN, SOX1, and PAX6) particularly in wild-type rather than the KDM6-DKO group, making the enhanced neuroectoderm differentiation phenotype in KDM6-DKO cells weakened as both showed high differentiation levels (Figures 6E,F). RNA analysis also indicated a similar conclusion (Figure 6G). These results provided functional evidence that the WNT signaling pathway contributed to the beneficial neuroectoderm differentiation due to the depletion of KDM6.

### KDM6 Inhibitor Improved Neuroectoderm Differentiation From Human ESCs

**FIGURE 7:**
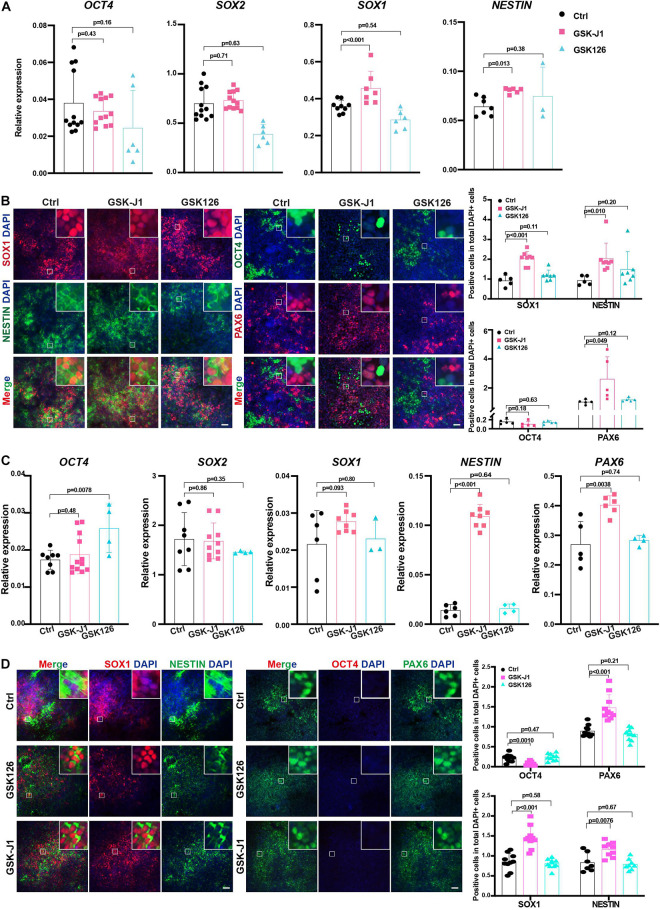
GSKJ1 improved neuroectoderm differentiation efficiency from human PSCs. **(A,B)** The RT-qPCR result **(A)** of the expression differences and immunofluorescence staining **(B)** of pluripotent or neural markers of HUES8 cells at differentiation day 7 upon adding GSKJ1 or GSK-126 or vehicle control. Scale bar = 50 μm. **(C,D)** The RT-qPCR result **(A)** of the expression differences and immunofluorescence staining **(B)** of pluripotent or neural markers of WTC cells at differentiation day 7 upon adding GSKJ1 or GSK-126 or vehicle control. Scale bar = 50 μm.

Our data showed that the genetic depletion of KDM6 facilitated neuroectoderm commitment; next, we applied the chemicals targeting KDM6 in human neuroectoderm differentiation. Since GSK-J1 is a well-recognized inhibitor of KDM6 while GSK126 is targeting H3K27 methyltransferase EZH2, we treated the neuroectoderm differentiation with GSK-J1 or GSK126 and then examined the efficiency by RT-qPCR and immunofluorescence. The results showed that the RNA levels of the pluripotent marker *OCT4* were not dramatically downregulated in the GSKJ1 group, but the RNA levels of the neural markers *SOX1* and *PAX6* were significantly increased (Figure 7A), whereas no beneficial effect was observed in the GSK126 group. Consistently, immunofluorescence staining also suggested that much more SOX1-, NESTIN-, and PAX6- positive cells emerged in the GSK-J1 group but no increase in the GSK126 group (Figure 7B). The quantification of the immunofluorescence showed the same results (Figure 7B). We further validated the effect of GSK-J1 in an iPSC line WTC and observed similar results (Figures 7C,D). Taken together, the KDM6 inhibitor GSK-J1 could significantly improve the neuroectoderm differentiation from human pluripotent stem cells.

## Discussion

In this study, we identified the role of KDM6A and KDM6B in human neuroectoderm commitment. We started to profile the epigenetic enzymes responsible for histone methylation and found that KDM6B exhibited the highest differential expression during neuroectoderm differentiation. By establishing knockout human ESC lines of KDM6, we demonstrated that KDM6 played an inhibitory role in neuroectoderm differentiation mainly through the WNT signal pathway. Moreover, we found that treatment with the KDM6 inhibitor GSK-J1 could greatly enhance the neuroectoderm differentiation from human ESCs, which might contribute to the generation of functional neural cells in regenerative medicine.

Very recently, there are three reports about the functional study of either KDM6A or KDM6B in human ESC maintenance and neural differentiation based on genetic manipulation. Two studies focused on KDM6A. Peng’s group reported that the interaction of 53BP1-KDM6A was necessary for neuron differentiation and development of cortical organoids from ESCs, and found that the interaction could promote KDM6A chromatin binding to initiate gene activation by removing H3K27me3 modification (Yang et al., 2019). Later, the same group further found that KDM6A suppressed gliogenesis by interacting with AP-1 (Xu et al., 2020). Another study reported that KDM6A controlled neuronal differentiation and dendritic morphogenesis after 40 days of differentiation (Tang et al., 2020). In addition, Shan et al. (2020) successfully established KDM6A/KDM6B double-knockout H1 ESC line and found that the KDM6 double-knockout ESCs showed no phenotype on either pluripotency maintenance or neural progenitor differentiation. Then they focused on the neuronal differentiation and found that KDM6 affected the identity and neuronal differentiation bias of neural progenitors (Shan et al., 2020). These studies together suggested an important role in further neuronal/glial differentiation from neural progenitors. Here we constructed KDM6 double-knockout HUES8 ESCs and found that KDM6-DKO cells could normally self-renew and keep the ability to differentiate into the three germ layers. More importantly, we observed that KDM6-DKO ESCs exhibited higher and more advanced neuroectoderm differentiation capacity. One possible reason might be the relatively natural differentiation method with not very high differentiation efficiency in our study, which makes the enhanced neural differentiation phenotype more evident. Indeed, Xu et al. (2020) reported that the KDM6A knockout ESCs exhibited a characteristic of neural progenitors to a certain extent, and it was much easier to direct the neural progenitor differentiation from KDM6A knockout ESCs, which supported our observation and collectively indicated that KDM6 was a barrier of neuroectoderm differentiation. Taken together, our study expanded the functional understanding of KDM6 in the entire neural lineage differentiation.

Epigenetic modifications play an important role in cell fate conversion, usually delaying or accelerating the process (Boland et al., 2014). Different epigenetic modifiers can function in the same process. The depletion of KDM5D or KDM6A both affected cardiomyocyte differentiation and failed to generate heart-like rhythmic contractions (Lee et al., 2012; Meyfour et al., 2019). In addition, the same epigenetic factor can function in different processes. For instance, knockdown of KDM5C could lead to facial deformities like small head cartilage and eye malformations (Kim et al., 2018); meanwhile, KDM5C played an important role in adipocyte differentiation (Link et al., 2020). More interestingly, the same epigenetic factor could exert a dual function in a different stage during development. For example, the H3K9 methyltransferase SUV39H1/2 acted as a barrier for reprogramming and preimplantation development (Zhang et al., 2018) but positively maintained the stemness in later epidermal lineage differentiation (Balmer et al., 2021). Here, our data together with other studies indicate that KDM6 plays a biphasic role in neurogenesis: KDM6 hinders neuroectoderm differentiation, the first step of neurogenesis, but is required for following neuronal and glial differentiation. Moreover, given that we previously reported that KDM6 could promote mesendoderm lineage differentiation from human ESCs (Jiang et al., 2013), KDM6 is able to function as a lineage switcher between neuroectoderm and mesendoderm, which provides more insights into the dual role of epigenetic regulation in cell fate determination.

Differentiation of diverse neurons from human PSCs provides a great promise for degenerative diseases in neural system, such as Huntington’s disease and Parkinson’s disease. A lot of attention has been paid to generate neural progenitors or neurons to transplant into animal models to treat neural degenerative diseases (Wang et al., 2007; Ziavra et al., 2012). In addition, chemicals with the ability to promote neurogenesis have been identified as pro-drugs, such as the potent topoisomerase I inhibitor SN-38 (Gutova et al., 2013) and EGb761 (Ren et al., 2019). Here, by looking into the biological function of KDM6, we identified that a WNT inhibitor and KDM6 inhibitor GSK-J1 could efficiently promote neuroectoderm commitment, which might facilitate the generation of functional neurons for cell therapy in nervous diseases.

## Data Availability Statement

The RNA-sequencing raw data have been uploaded in GEO with accession no. GSE172312.

## Author Contributions

WJ conceived the project, obtained the funding support with YJ, and designed the experiment together with YM. YM established the KDM6 knockout ESCs with help from SD. YM performed the neuroectoderm differentiation assay together with TZ and SD. TZ analyzed the transcriptome data. RZ performed the endoderm differentiation assay with RL and helped in the figure preparation. JY performed the Western blot together with YM. YM and TZ drafted. WJ finalized the manuscript. All authors contributed to and approved the final manuscript.

## Conflict of Interest

The authors declare that the research was conducted in the absence of any commercial or financial relationships that could be construed as a potential conflict of interest.

## Publisher’s Note

All claims expressed in this article are solely those of the authors and do not necessarily represent those of their affiliated organizations, or those of the publisher, the editors and the reviewers. Any product that may be evaluated in this article, or claim that may be made by its manufacturer, is not guaranteed or endorsed by the publisher.

## Footnotes

1
http://crispr.mit.edu/

2
http://geneontology.org/

3
http://stemcell.libd.org/scb/

## Supplementary Material

Supplementary Figure 1 The expression dynamics of KDM6B and KDM6A during neural differentiation based on LIBD Stem Cell Browser. The diagram is from the website (http://stemcell.libd.org/scb/) with minor modifications.

Supplementary Table 1 TPM analysis of RNA-seq data.

## References

[B1] Balmer P. Hariton W. V. J. Sayar B. S. Jagannathan V. Galichet A. Leeb T. (2021). SUV39H2 epigenetic silencing controls fate conversion of epidermal stem and progenitor cells. *J. Cell Biol.* 220:e201908178. 10.1083/jcb.201908178 PMC7898489 33604655

[B2] Bernstein B. E. Mikkelsen T. S. Xie X. Kamal M. Huebert D. J. Cuff J. (2006). A bivalent chromatin structure marks key developmental genes in embryonic stem cells. *Cell* 125 315–326. 10.1016/j.cell.2006.02.041 16630819

[B3] Blauwkamp T. A. Nigam S. Ardehali R. Weissman I. L. Nusse R. (2012). Endogenous Wnt signalling in human embryonic stem cells generates an equilibrium of distinct lineage-specified progenitors. *Nat. Commun.* 3:1070. 10.1038/ncomms2064 PMC3657997 22990866

[B4] Boland M. J. Nazor K. L. Loring J. F. (2014). Epigenetic regulation of pluripotency and differentiation. *Circ. Res.* 115 311–324. 10.1161/circresaha.115.301517 24989490 PMC4229506

[B5] Burgold T. Voituron N. Caganova M. Tripathi P. P. Menuet C. Tusi B. K. (2012). The H3K27 demethylase JMJD3 is required for maintenance of the embryonic respiratory neuronal network, neonatal breathing, and survival. *Cell Rep.* 2 1244–1258. 10.1016/j.celrep.2012.09.013 23103168

[B6] Chailangkarn T. Trujillo C. A. Freitas B. C. Hrvoj-Mihic B. Herai R. H. Yu D. X. (2016). A human neurodevelopmental model for Williams syndrome. *Nature* 536 338–343. 27509850 10.1038/nature19067 PMC4995142

[B7] Chambers S. M. Fasano C. A. Papapetrou E. P. Tomishima M. Sadelain M. Studer L. (2009). Highly efficient neural conversion of human ES and iPS cells by dual inhibition of SMAD signaling. *Nat. Biotechnol.* 27 275–280. 10.1038/nbt.1529 19252484 PMC2756723

[B8] Chambers S. M. Mica Y. Studer L. Tomishima M. J. (2011). Converting human pluripotent stem cells to neural tissue and neurons to model neurodegeneration. *Methods Mol. Biol.* 793 87–97. 10.1007/978-1-61779-328-8_6 21913095

[B9] Faundes V. Newman W. G. Bernardini L. Canham N. Clayton-Smith J. Dallapiccola B. (2018). Histone lysine methylases and demethylases in the landscape of human developmental disorders. *Am. J. Hum. Genet.* 102 175–187. 29276005 10.1016/j.ajhg.2017.11.013 PMC5778085

[B10] Fujihara Y. Ikawa M. (2014). CRISPR/Cas9-based genome editing in mice by single plasmid injection. *Methods Enzymol.* 546 319–336. 10.1016/b978-0-12-801185-0.00015-5 25398347

[B11] Gazova I. Lengeling A. Summers K. M. (2019). Lysine demethylases KDM6A and UTY: the X and Y of histone demethylation. *Mol. Genet. Metab.* 127 31–44. 10.1016/j.ymgme.2019.04.012 31097364

[B12] Gutova M. Shackleford G. M. Khankaldyyan V. Herrmann K. A. Shi X. H. Mittelholtz K. (2013). Neural stem cell-mediated CE/CPT-11 enzyme/prodrug therapy in transgenic mouse model of intracerebellar medulloblastoma. *Gene Ther.* 20 143–150. 10.1038/gt.2012.12 22402322 PMC4149468

[B13] Imagawa E. Albuquerque E. V. A. Isidor B. Mitsuhashi S. Mizuguchi T. Miyatake S. (2018). Novel SUZ12 mutations in Weaver-like syndrome. *Clin. Genet.* 94 461–466. 10.1111/cge.13415 30019515

[B14] Imagawa E. Higashimoto K. Sakai Y. Numakura C. Okamoto N. Matsunaga S. (2017). Mutations in genes encoding polycomb repressive complex 2 subunits cause Weaver syndrome. *Hum. Mutat.* 38 637–648. 10.1002/humu.23200 28229514

[B15] Irmak D. Fatima A. Gutierrez-Garcia R. Rinschen M. M. Wagle P. Altmuller J. (2018). Mechanism suppressing H3K9 trimethylation in pluripotent stem cells and its demise by polyQ-expanded huntingtin mutations. *Hum. Mol. Genet.* 27 4117–4134. 30452683 10.1093/hmg/ddy304

[B16] Jiang W. Wang J. Zhang Y. (2013). Histone H3K27me3 demethylases KDM6A and KDM6B modulate definitive endoderm differentiation from human ESCs by regulating WNT signaling pathway. *Cell Res.* 23 122–130. 10.1038/cr.2012.119 22907667 PMC3541667

[B17] Jones S. E. Olsen L. Gajhede M. (2018). Structural basis of histone demethylase KDM6B histone 3 lysine 27 specificity. *Biochemistry* 57 585–592. 10.1021/acs.biochem.7b01152 29220567

[B18] Kim T. W. Koo S. Y. Studer L. (2020). Pluripotent stem cell therapies for Parkinson disease: present challenges and future opportunities. *Front. Cell Dev. Biol.* 8:729. 10.3389/fcell.2020.00729 32903681 PMC7438741

[B19] Kim Y. Jeong Y. Kwon K. Ismail T. Lee H. K. Kim C. (2018). Physiological effects of KDM5C on neural crest migration and eye formation during vertebrate development. *Epigenet. Chromatin* 11:72. 10.1186/s13072-018-0241-x PMC6282277 30522514

[B20] Kimelman D. Xu W. (2006). beta-catenin destruction complex: insights and questions from a structural perspective. *Oncogene* 25 7482–7491. 17143292 10.1038/sj.onc.1210055

[B21] Laugesen A. Hojfeldt J. W. Helin K. (2019). Molecular mechanisms directing PRC2 recruitment and H3K27 methylation. *Mol. Cell* 74 8–18. 10.1016/j.molcel.2019.03.011 30951652 PMC6452890

[B22] Lee S. Lee J. W. Lee S. K. (2012). UTX, a histone H3-lysine 27 demethylase, acts as a critical switch to activate the cardiac developmental program. *Dev. Cell* 22 25–37. 10.1016/j.devcel.2011.11.009 22192413 PMC4111644

[B23] Link J. C. Wiese C. B. Chen X. Avetisyan R. Ronquillo E. Ma F. (2020). chromosome dosage of histone demethylase KDM5C determines sex differences in adiposity. *J. Clin. Invest.* 130 5688–5702. 10.1172/jci140223 32701509 PMC7598065

[B24] Meyfour A. Pahlavan S. Ansari H. Baharvand H. Salekdeh G. H. (2019). Down-Regulation of a male-specific H3K4 demethylase, KDM5D, impairs cardiomyocyte differentiation. *J. Proteome Res.* 18 4277–4282. 10.1021/acs.jproteome.9b00395 31560558

[B25] Miyaoka Y. Chan A. H. Judge L. M. Yoo J. Huang M. Nguyen T. D. (2014). Isolation of single-base genome-edited human iPS cells without antibiotic selection. *Nat. Methods* 11 291–293. 10.1038/nmeth.2840 24509632 PMC4063274

[B26] Moya N. Cutts J. Gaasterland T. Willert K. Brafman D. A. (2014). Endogenous WNT signaling regulates hPSC-derived neural progenitor cell heterogeneity and specifies their regional identity. *Stem Cell Rep.* 3 1015–1028. 10.1016/j.stemcr.2014.10.004 PMC4264562 25458891

[B27] Nakano T. Ando S. Takata N. Kawada M. Muguruma K. Sekiguchi K. (2012). Self-formation of optic cups and storable stratified neural retina from human ESCs. *Cell Stem Cell* 10 771–785. 10.1016/j.stem.2012.05.009 22704518

[B28] Podobinska M. Szablowska-Gadomska I. Augustyniak J. Sandvig I. Sandvig A. Buzanska L. (2017). Epigenetic modulation of stem cells in neurodevelopment: the role of methylation and acetylation. *Front. Cell. Neurosci.* 11:23. 10.3389/fncel.2017.00023 28223921 PMC5293809

[B29] Ren C. Ji Y. Q. Liu H. Wang Z. Wang J. H. Zhang C. Y. (2019). Effects of Ginkgo biloba extract EGb761 on neural differentiation of stem cells offer new hope for neurological disease treatment. *Neural Regen. Res.* 14 1152–1157. 10.4103/1673-5374.251191 30804240 PMC6425836

[B30] Sengoku T. Yokoyama S. (2011). Structural basis for histone H3 Lys 27 demethylation by UTX/KDM6A. *Genes Dev.* 25 2266–2277. 10.1101/gad.172296.111 22002947 PMC3219231

[B31] Shalem O. Sanjana N. E. Hartenian E. Shi X. Scott D. A. Mikkelson T. (2014). Genome-scale CRISPR-Cas9 knockout screening in human cells. *Science* 343 84–87. 24336571 10.1126/science.1247005 PMC4089965

[B32] Shan Y. Zhang Y. Zhao Y. Wang T. Zhang J. Yao J. (2020). JMJD3 and UTX determine fidelity and lineage specification of human neural progenitor cells. *Nat. Commun.* 11:382. 10.1038/s41467-019-14028-x PMC6971254 31959746

[B33] Shpargel K. B. Sengoku T. Yokoyama S. Magnuson T. (2012). UTX and UTY demonstrate histone demethylase-independent function in mouse embryonic development. *PLoS Genet.* 8:e1002964. 10.1371/journal.pgen.1002964 23028370 PMC3459986

[B34] Tang G. B. Zeng Y. Q. Liu P. P. Mi T. W. Zhang S. F. Dai S. K. (2017). The histone H3K27 demethylase UTX regulates synaptic plasticity and cognitive behaviors in mice. *Front. Mol. Neurosci.* 10:267. 10.3389/fnmol.2017.00267 28970783 PMC5609596

[B35] Tang Q. Y. Zhang S. F. Dai S. K. Liu C. Wang Y. Y. Du H. Z. (2020). UTX regulates human neural differentiation and dendritic morphology by resolving bivalent promoters. *Stem Cell Rep.* 15 439–453. 10.1016/j.stemcr.2020.06.015 32679064 PMC7419705

[B36] Topol A. Tran N. N. Brennand K. J. (2015). A guide to generating and using hiPSC derived NPCs for the study of neurological diseases. *J. Vis. Exp.* 96:e52495. 10.3791/52495 PMC4354663 25742222

[B37] Tran N. Broun A. Ge K. (2020). Lysine demethylase KDM6A in differentiation, development, and cancer. *Mol. Cell. Biol.* 40:e00341-20. 10.1128/MCB.00341-20 PMC7523656 32817139

[B38] Wang L. Martin D. R. Baker H. J. Zinn K. R. Kappes J. C. Ding H. (2007). Neural progenitor cell transplantation and imaging in a large animal model. *Neurosci. Res.* 59 327–340. 10.1016/j.neures.2007.08.011 17897743

[B39] Wang T. Wei J. J. Sabatini D. M. Lander E. S. (2014). Genetic screens in human cells using the CRISPR-Cas9 system. *Science* 343 80–84. 10.1126/science.1246981 24336569 PMC3972032

[B40] Xu B. Mulvey B. Salie M. Yang X. Matsui Y. Nityanandam A. (2020). UTX/KDM6A suppresses AP-1 and a gliogenesis program during neural differentiation of human pluripotent stem cells. *Epigenet. Chromatin* 13:38. 10.1186/s13072-020-00359-3 PMC7519529 32977832

[B41] Yang J. Lu P. Li M. Yan C. Zhang T. Jiang W. (2020). GATA6-AS1 regulates GATA6 expression to modulate human endoderm differentiation. *Stem Cell Rep.* 15 694–705. 10.1016/j.stemcr.2020.07.014 32795420 PMC7486217

[B42] Yang X. Xu B. Mulvey B. Evans M. Jordan S. Wang Y. D. (2019). Differentiation of human pluripotent stem cells into neurons or cortical organoids requires transcriptional co-regulation by UTX and 53BP1. *Nat. Neurosci.* 22 362–373. 10.1038/s41593-018-0328-5 30718900 PMC6511450

[B43] Zhang S. C. Wernig M. I Duncan D. Brustle O. Thomson J. A. (2001). In vitro differentiation of transplantable neural precursors from human embryonic stem cells. *Nat. Biotechnol.* 19 1129–1133. 10.1038/nbt1201-1129 11731781

[B44] Zhang Y. L. Zhao L. W. Zhang J. Le R. Ji S. Y. Chen C. (2018). DCAF13 promotes pluripotency by negatively regulating SUV39H1 stability during early embryonic development. *EMBO J.* 37:e98981. 10.15252/embj.201898981 PMC6138440 30111536

[B45] Ziavra D. Makri G. Giompres P. Taraviras S. Thomaidou D. Matsas R. (2012). Neural stem cells transplanted in a mouse model of Parkinson’s disease differentiate to neuronal phenotypes and reduce rotational deficit. *CNS Neurol. Disord. Drug Targets* 11 829–835. 10.2174/1871527311201070829 23131156

