# Supplementary figures and images for: Depletion of Demethylase KDM6 Enhances Early Neuroectoderm Commitment of Human PSCs
Source: Front Cell Dev Biol. 2021 Sep 8;9:702462. doi: 10.3389/fcell.2021.702462 (PMC8455897; doi:10.3389/fcell.2021.702462)

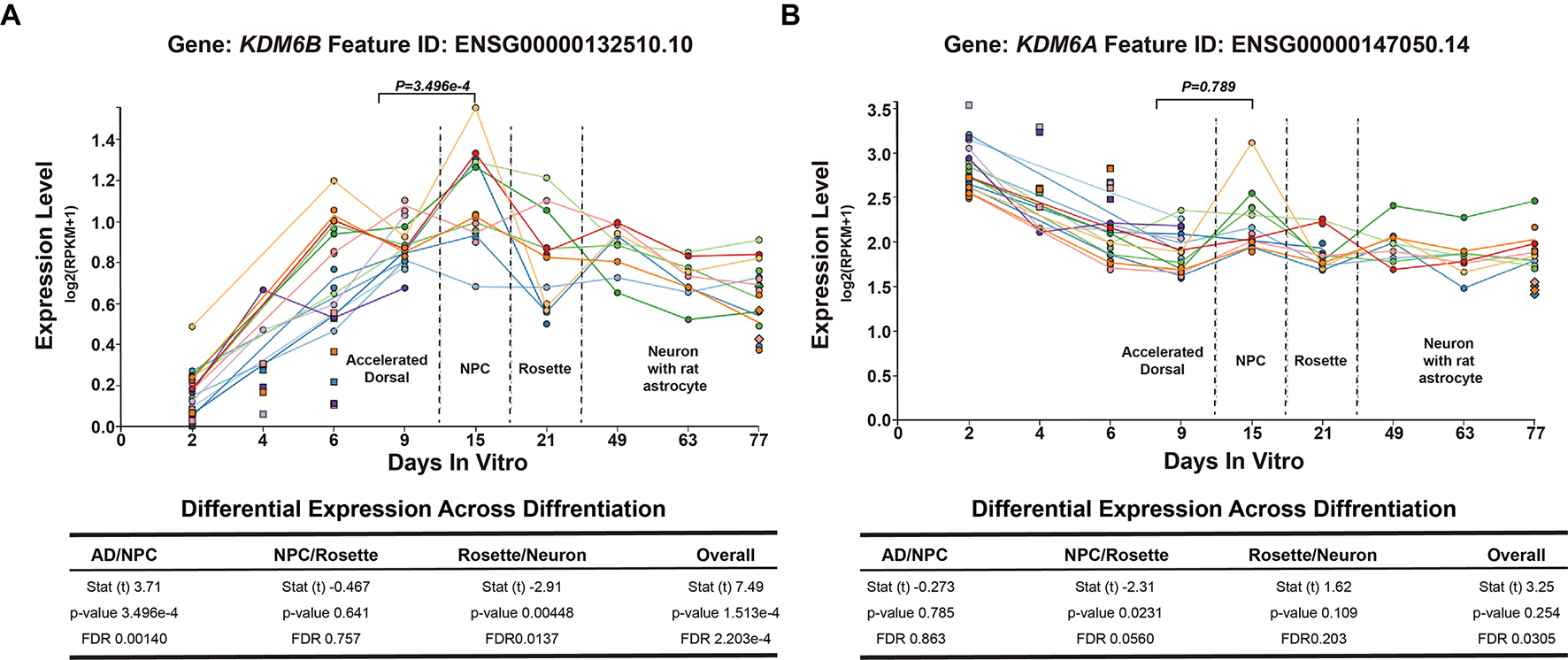

Supplement: Supplementary Figure 1 — The expression dynamics of KDM6B and KDM6A during neural differentiation based on LIBD Stem Cell Browser. The diagram is from the website (http://stemcell.libd.org/scb/) with minor modifications. [file Image_1.JPEG]
